# Traumatic abdominal wall hernias: A 15-year single-center experience in diagnosis and management

**DOI:** 10.1007/s00595-025-03179-8

**Published:** 2025-11-19

**Authors:** Hye Young Woo, Kyoungwon Jung

**Affiliations:** 1 Division of Trauma Surgery, Department of Surgery, Ajou University School of Medicine, 164 World cup-ro, Yeongtong-gu, Suwon, 16499 Republic of Korea; 2 Ajou University Hospital Gyeonggi South Regional Trauma Center, Suwon, Republic of Korea

**Keywords:** Trauma and injuries, Blunt abdominal trauma, Abdominal wall injury, Abdominal wall hernia, Hernia repair

## Abstract

**Purpose:**

Traumatic abdominal wall hernia (TAWH) is a rare injury caused by high-energy blunt trauma. Its diagnosis is often missed, and the current management strategies remain inconsistent. This study aimed to describe the clinical characteristics, diagnostic challenges, and surgical outcomes of TAWH based on a 15-year experience at a high-volume trauma center in Korea.

**Methods:**

We retrospectively reviewed the records of patients diagnosed with TAWH between 2010 and 2024. The data included demographics, injury mechanisms, hernia features, surgical timing, and outcomes.

**Results:**

Of 17,852 patients with blunt abdominal trauma, 28 (0.16%) were diagnosed with TAWH. Although CT identified most hernias (96.4%), only 48.1% were noted in official radiology reports. Common hernia locations were lumbar (38.7%) and anterior (29.0%). Associated intra-abdominal injuries were present in 89.3% of cases. Surgical repair was performed in 22 patients, with 90.9% undergoing early repair during the index admission. Two patients underwent delayed mesh repair, without recurrence. Overall recurrence and surgical site infection rates were both 18.2%.

**Conclusion:**

TAWH remains under-recognized despite its strong association with high-energy trauma and clear CT detectability. Findings from this long-term single-center study support early repair when feasible and selective delayed mesh repair is performed in appropriate patients, underscoring the need for greater awareness among trauma providers.

**Supplementary Information:**

The online version contains supplementary material available at 10.1007/s00595-025-03179-8.

## Introduction

Traumatic abdominal wall hernias (TAWH) are clinically significant injuries characterized by disruption of the abdominal musculature and fascia without penetrating skin wounds, typically resulting from high-energy blunt trauma [1–3]. Epidemiological studies have reported that TAWH is relatively uncommon, occurring in 0.17% to 0.9% of blunt trauma admissions [1, 4, 5]. Their diagnosis typically relies on clinical examinations in addition to radiologic imaging studies. The increasing availability and routine use of computed tomography (CT) in trauma settings have enhanced diagnostic accuracy, contributing to an increase in the reported incidence of TAWH [2, 5–7]. Its predominant mechanisms include motor vehicle collisions, motorcycle crashes, handlebar injuries, and falls from height, all of which generate significant blunt and shearing forces sufficient to disrupt the abdominal wall integrity [8]. TAWHs are not merely isolated herniations but are frequently accompanied by significant intra-abdominal injuries, such as bowel perforation, mesenteric injury, and solid organ lacerations [4, 7]. These injuries significantly influence patient morbidity and management decisions [4, 5, 7, 9, 10]. The timing and methods of hernia repair remain controversial, with debates over immediate vs. delayed repair and primary closure vs. mesh use [2, 4, 11–13]. Given the paucity of large, prospective randomized trials, clinical guidelines remain inconsistent, with management often guided by surgeon preference and individual institutional practices. Consequently, further detailed clinical analyses and reporting are essential to better delineate the patient characteristics, associated injuries, complications, and outcomes. This study aimed to systematically evaluate TAWH patients and provide data that may inform evidence-based clinical guidelines on their diagnosis and management.

## Methods

This retrospective study prospectively analyzed data collected from the Ajou University Hospital Trauma Registry for patients with TAWH (January 2010 to December 2024). Ajou University Hospital is a tertiary educational medical institution located in Suwon City, South Korea. It operates as a regional trauma center, equivalent to a Level 1 trauma center in the United States. The registry was searched for free text “hernia.” Patients admitted with a clinical or radiological diagnosis of TAWH were included in the study. Patients with penetrating injuries, pre-existing hernias, incisional hernias, or diaphragmatic hernias were excluded.

### Variables evaluated

Patients’ electronic medical records were reviewed, and data on (1) patient demographics and anthropometric data (age, sex, height, weight, body mass index [BMI], skeletal muscle mass [SMM], comorbidities, intensive care unit [ICU] admission, length of stay [LOS], and mortality); (2) patient characteristics of injury (mechanism of injury, Injury Severity Score [ISS], and associated injuries); (3) clinical and lesion characteristics of TAWH (abdominal signs, CT findings, and lesion characteristics including laterality, number, anatomical location, grade, herniated contents, defect size); and (4) management-related data (timing of repair, surgical approach, incision, specific repair technique, and postoperative complications) were collected.

### Evaluation of skeletal muscle mass

Axial CT images of each patient at the level of the inferior endplate of the third lumbar vertebra (L3) were analyzed. Body composition was assessed using a deep learning system-based fully convolutional network automatic segmentation technique (AID-UTM, iAID Inc., Seoul, Korea) [14]. The software automatically delineated anatomical boundaries and calculated the skeletal muscle area (SMA, cm^2^), including the psoas major, paraspinals, transversus abdominis, rectus abdominis, quadratus lumborum, and internal and external oblique muscles, using predetermined thresholds (−29 to 150 Hounsfield units) (Supplementary Fig. 1). The skeletal muscle index (SMI) was calculated by dividing SMA (cm²) by the square of the patient’s height in meters (height², m²), expressed as cm²/m². Two Korean-specific cutoff values for sarcopenia were applied: conventional thresholds of 49 cm²/m² for males and 31 cm²/m² for females [15, 16], and updated reference values of 40.96 cm²/m² for males and 30.60 cm²/m² for females based on the 5th percentile of L3 SMI in healthy Korean adults [17].

### Grading, classification, and definition

All CT scans were retrospectively and independently reviewed by two board-certified trauma surgeons experienced in radiologic evaluation and management of traumatic abdominal injuries. The reviewers were blinded to the official radiology reports but had access to relevant clinical histories to replicate real-world trauma assessments. Discrepancies in interpretation were resolved by joint consensus, enhancing diagnostic reliability and minimizing observer bias. All TAWH diagnoses in this study were confirmed using CT imaging performed prior to surgical intervention, with no cases diagnosed solely intraoperatively.

We applied the standardized grading system developed by Dennis et al. [3] to objectively classify abdominal wall injuries on CT imaging: grade I, subcutaneous tissue contusion; grade II, abdominal wall muscle hematoma; grade III, single abdominal wall muscle disruption; grade IV, complete abdominal wall muscle disruption; grade V, complete abdominal wall muscle disruption with herniation of abdominal contents; and grade VI, open herniation (evisceration). Patients with grade I or II injuries, representing superficial contusions or hematomas without significant structural disruption, were excluded because these do not constitute true abdominal wall defects. Only grade ≥ III injuries (grades III–VI), which involve definitive muscular or fascial disruption, were included in the analysis. Grade VI injuries, despite their distinct presentation as open evisceration, were intentionally included within the TAWH spectrum because of their shared traumatic mechanism, clinical relevance, and similar management considerations as grade V injuries, consistent with prior studies [3, 7, 31].

Hernias involving the anterior abdominal wall were designated as “anterior” and further subclassified into two types: rectus hernias with defects of the rectus abdominis and Spigelian hernias defined by defects located along the Spigelian aponeurosis. Flank hernias involve the oblique muscle group, including the internal and external obliques and the transversus abdominis. Lumbar hernias were defined as posterolateral defects that were confined to the lumbar triangle. Hernias located in the groin region were classified as “inguinal.” Intercostal hernias are characterized by defects adjacent to the ribs.

Patients with TAWH who underwent operative management were categorized into the early and late repair groups based on the timing of surgical intervention. Early repair was defined as surgical repair performed during the index hospitalization, whereas late repair was defined as procedures conducted after discharge from the index admission. Furthermore, early repairs were sub-classified by timing: emergency repair was defined as surgery performed within 24 h of presentation, while delayed or elective repair referred to procedures performed urgently or electively beyond 24 h but still during the same hospitalization.

### Statistical analysis

Data were analyzed using SPSS (ver. 29.0; IBM Corp., Armonk, NY, USA). Descriptive analysis was performed to analyze the data using exact frequencies, percentages, medians with interquartile ranges (IQR), and means with standard deviations (SD). To assess differences in clinical and hernia-related characteristics according to the mechanism of injury, one-way analysis of variance (ANOVA) was used for parametric variables, and the Kruskal–Wallis test was used for non-parametric variables. Categorical variables were analyzed using Pearson’s chi-square or Fisher’s exact test. Fisher’s exact test was also performed in subgroup analyses to assess the influence of intra-abdominal contamination on surgical site infection (SSI) rates in early surgical repair, to evaluate its potential impact on mesh placement decisions, and to compare hernia recurrence rates between different repair techniques. *P* values of < 0.05 were considered to indicate statistical significance.

## Results

**Fig. 1:**
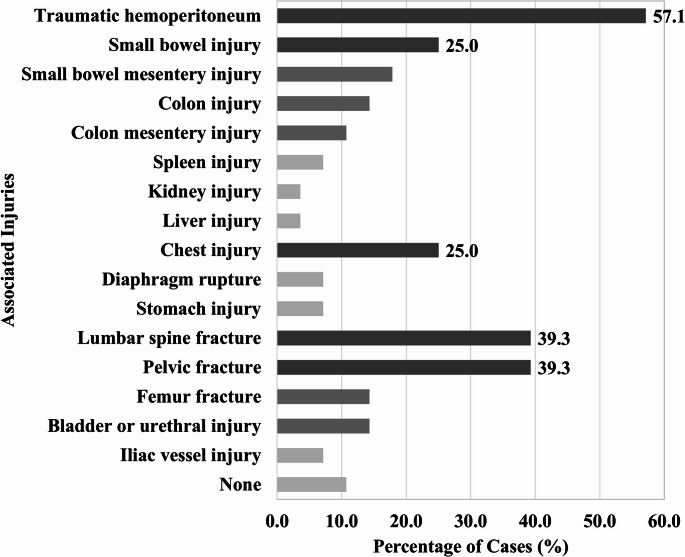
Injuries associated with traumatic abdominal wall hernias

From January 2011 to December 2024, 19,306 patients with trauma were admitted, 17,852 sustained blunt abdominal traumas, and 28 met the inclusion criteria, representing 0.16% incidence among patients with blunt abdominal trauma. The patient demographics are described in Table 1. The mean age was 52.2 ± 16.6 years, with the majority being younger than 65 years of age (78.6%). The mean BMI was 24.5 ± 3.6 kg/m^2^, which remained within the normal distribution [18]. Based on two Korean-specific SMI cutoff values for sarcopenia, the proportion of patients with decreased skeletal muscle mass was relatively low at 19.4% and 6.5%, respectively. Among patients with TAWH, 75.0% required ICU admission. The median ISS was 17.5 (IQR, 8.8–27.0). Associated injuries were observed in 25 patients (89.3%). Traumatic hemoperitoneum was the most frequent (57.1%). Lumbar spine and pelvic fractures were each observed in 39.3% of cases, and small bowel and thoracic injuries were commonly identified (Fig. 1).

**Table 1:** Demographics of patients with traumatic abdominal wall hernia

Variables	Patients (*n* = 28)
Age (years), mean ± SD	52.2 ± 16.6
Elderly (< 65 years), n (%)	22 (78.6)
Sex, male, n (%)	18 (64.3)
Height (cm), mean ± SD	165.6 ± 9.8
Weight (kg), mean ± SD	67.6 ± 14.1
Body mass index (kg/m^2^), mean ± SD	24.5 ± 3.6
Skeletal muscle mass (cm^2^), mean ± SD	140.5 ± 42.6
Skeletal muscle index (cm^2^/m^2^), mean ± SD	51.3 ± 15.1
Decreased skeletal muscle mass using prior Korean cut-off, n (%)	6 (19.4)
Decreased skeletal muscle mass using new Korean cut-off, n (%)	2 (6.5)
Comorbidities, n (%)	
Hypertension	9 (32.1)
Diabetes mellitus	1 (3.6)
COPD or asthma	0 (0.0)
Coronary artery disease	2 (7.1)
Cerebrovascular disease	2 (7.1)
Grand-multipara	2 (7.1)
Multipara	7 (25.0)
Previous abdominal operation history	5 (17.9)
Previous abdominal trauma history	1 (3.6)
Current-smoker	7 (25.0)
Current-drinker	13 (46.4)
ICU admission, n (%)	21 (75.0)
ICU length of stay (days), mean ± SD	10.5 ± 21.1
Total length of stay (days), mean ± SD	39.8 ± 51.4
Mortality, n (%)	1 (3.6)
Discharge status, n (%)	
Home	10 (35.7)
Transfer	17 (60.7)
Death	1 (3.6)
Follow-up duration (month)	
mean ± SD	11.3 ± 15.5
median (IQR)	4.3 (0.5–14.1)
ISS, median (IQR)	17.5 (8.8–27.0)
AIS head	0 (0.0–0.0)
AIS chest	0 (0.0–3.0)
AIS abdomen	2.5 (2.0–3.0)
AIS spine	0 (0.0–0.0)
AIS extremity or pelvis girdle	2 (0.0–3.3)
AIS external	1 (0.0–1.0)
Associated injuries, n (%)	25 (89.3)

Abbreviations: SD, standard deviation; COPD, chronic obstructive pulmonary disease; ICU, intensive care unit; IQR, interquartile range; ISS, Injury Severity Score; AIS: Abbreviated Injury Scale

**Fig. 2:**
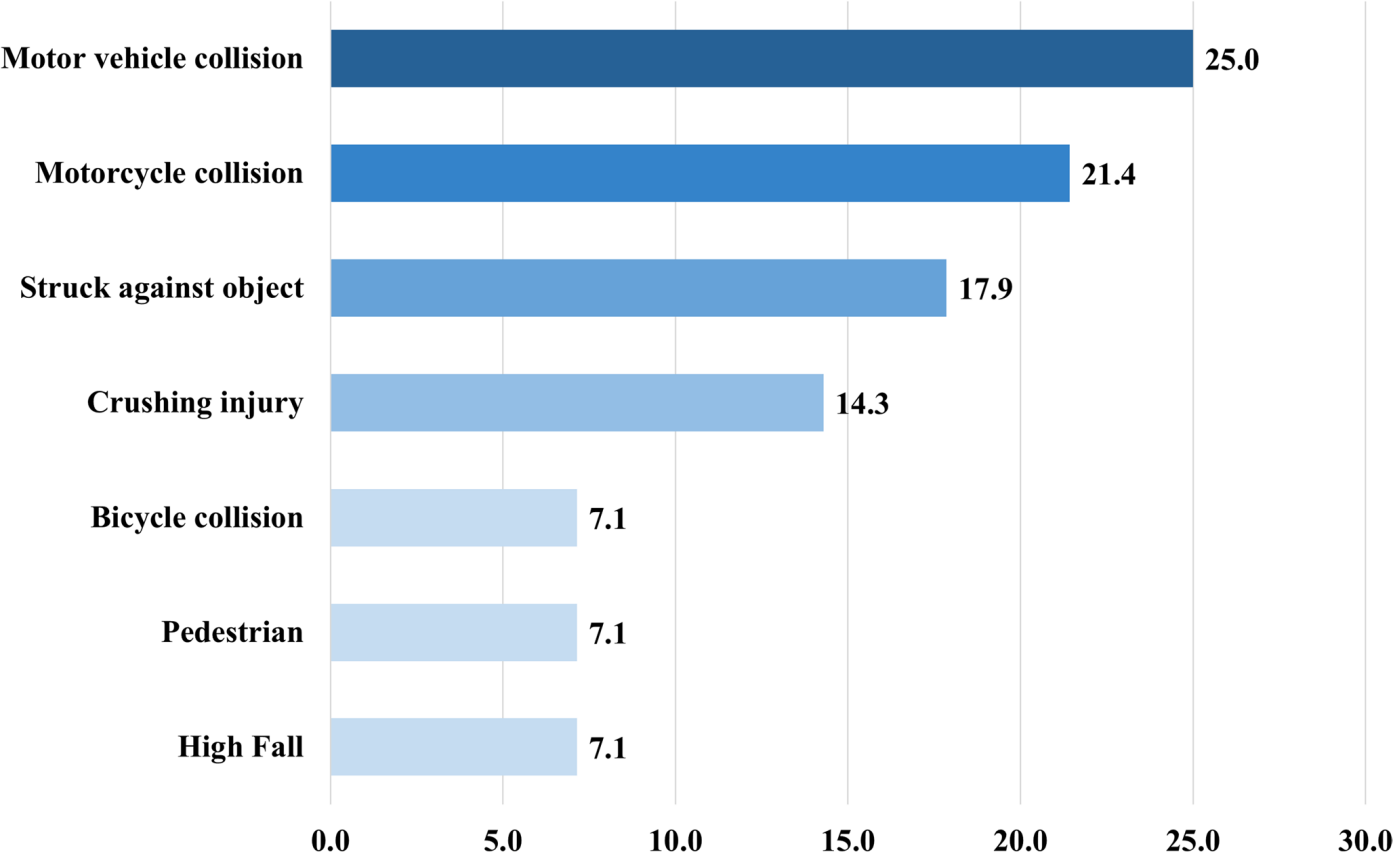
Mechanisms of injury in traumatic abdominal wall hernia

**Fig. 3:**
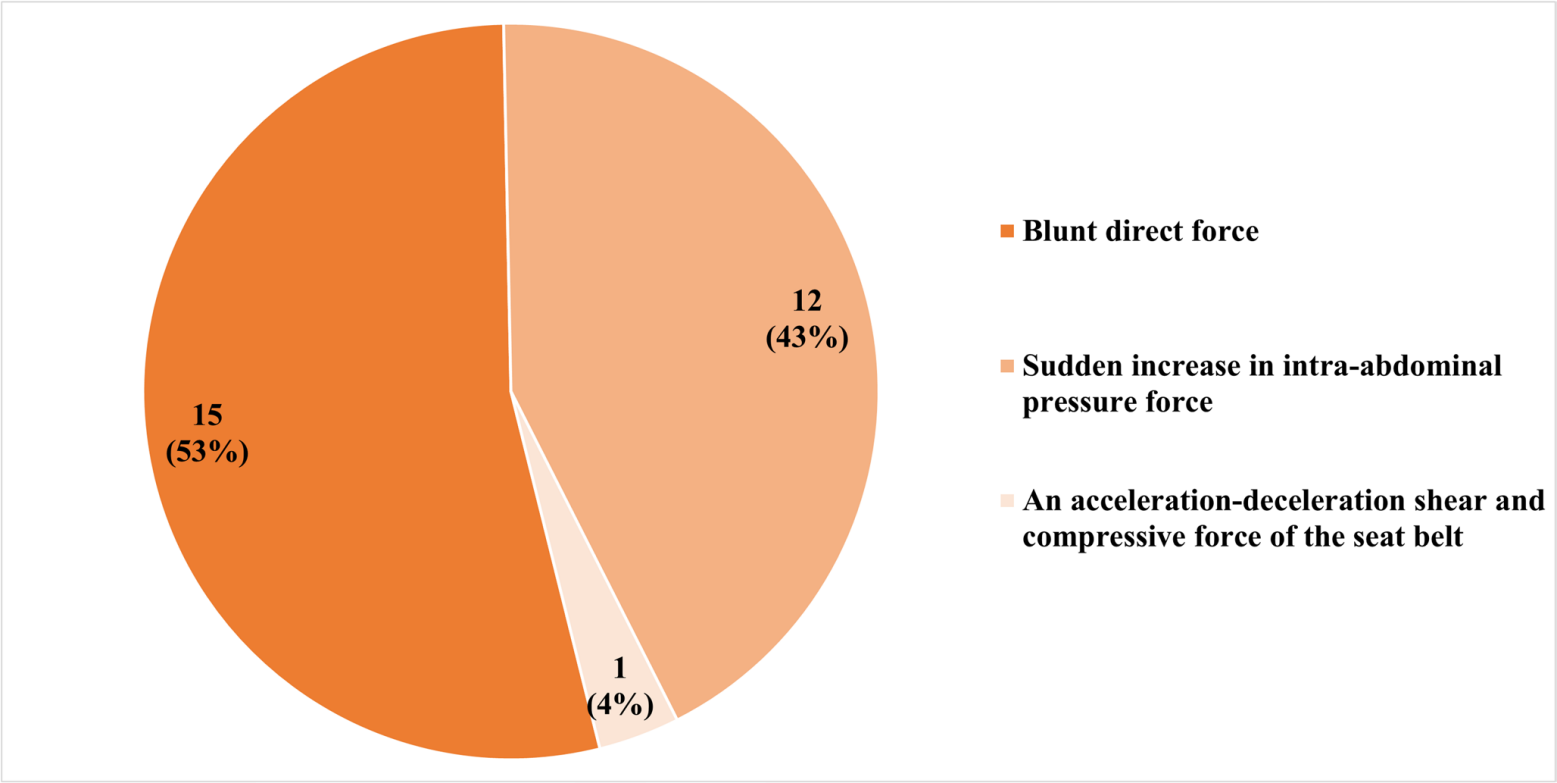
Mechanisms of energy impact in traumatic abdominal wall hernia

Motor vehicle collisions (MVCs) were the most common mechanism of injury (25.0%), then motorcycle collisions (21.4%), being struck by an object (17.9%), crush injuries (14.3%), bicycle collisions (7.1%), pedestrian impacts (7.1%), and high falls (7.1%) (Fig. 2). Based on the direction and nature of force applied [19], injuries were classified as resulting from direct blunt impact, which represents the typical handle bar injury, the animal gore, or any blunt direct force to the abdominal wall (53.6%), sudden increase in intra-abdominal pressure such as MVC, high fall, or high-speed motorcycle collision (42.9%), or seatbelt-associated compressive force (3.6%) (Fig. 3). Patients presented with a range of abdominal symptoms suggestive of TAWH, with abdominal pain being the most common complaint (*n* = 18, 64.3%) (Supplementary Table 1). Physical findings, such as abdominal wall contusion (*n* = 8, 28.6%) or bulging (*n* = 5, 17.9%), were observed in several cases. TAWH findings were identified on CT imaging in all but one patient; however, the diagnosis was explicitly mentioned in the official radiology report in only 48.1% of cases.

The anatomical distribution of TAWH included anterior hernias in nine patients (29.0%), lumbar hernias in 12 (38.7%), flank hernias in three (9.7%), inguinal hernias in five (16.1%), and intercostal hernias in two (6.5%) (Supplementary Table 2). According to the Dennis grading system, Grade V was the most commonly observed injury type (*n* = 20, 64.5%), followed by Grade IV (*n* = 9, 29.0%). One case of Grade III hernia and one case of Grade VI evisceration were identified.

The group experiencing a sudden increase in intra-abdominal pressure had a significantly higher ISS than the other groups (12.1 ± 10.3 vs. 30.3 ± 13.2 vs. 12.0, *p* = 0.002). The blunt direct force group had a lower proportion of patients with associated injuries (80.0% vs. 100.0% vs. 100.0%, *p* = 0.132) and underwent surgical repair less frequently (66.7% vs. 75.0% vs. 100.0%, *p* = 0.634) in comparison to the other groups, without a statistically significant difference. Anterior hernias were the most common type in the blunt direct force group (*n* = 6, 37.5%), followed by inguinal hernias (*n* = 4, 25.0%), and lumbar hernias (*n* = 9, 64.3%) were most frequently observed in the group with sudden increases in intra-abdominal pressure. However, the distribution of hernia types did not differ significantly among the groups (*p* = 0.270) (Table 2).

**Table 2:** Clinical and lesion characteristics of traumatic abdominal wall hernia according to mechanisms of energy impact

Patients (*n* = 28)	Blunt direct force (*n* = 15)	Sudden increase in intra-abdominal pressure (*n* = 12)	Seatbelt-associated shear force (*n* = 1)	*p*-value
Total LOS	12.0 (6.0 - 19.0)	39.5.0 (16.0 - 76.5)	13.0	0.246
ICU LOS	2.0 (2.0 - 7.0)	6.0 (2.75 - 15.75)	6.0	0.054
ISS	12.1 ± 10.3	30.3 ± 13.2	12.0	0.002
Associated injuries	12 (80.0)	12 (100.0)	1 (100.0)	0.132
Operative repair for TAWH	10 (66.7)	9 (75.0)	1 (100.0)	0.634
Underwent operation	11 (73.3)	11 (91.7)	1 (100.0)	0.369
Immediate laparotomy	9 (81.8)	8 (72.7)	0 (0.0)	0.214
Damage control surgery	2 (18.2)	2 (18.2)	0 (0.0)	0.822
Cases (*n* = 31)	1 (*n* = 16)	2 (*n* = 14)	3 (*n* = 1)	*p*-value
Defect size (cm)	4.5 ± 2.7	6.3 ± 4.6	2.0	0.297
Locations				0.270
Anterior	6 (37.5)	2 (14.3)	1 (100.0)	
Flank	2 (12.5)	1 (7.1)	0 (0.0)	
Lumbar	3 (18.8)	9 (64.3)	0 (0.0)	
Inguinal	4 (25.0)	1 (7.1)	0 (0.0)	
Intercostal	1 (6.3)	1 (7.1)	0 (0.0)	
Grade*				0.717
III	1 (6.3)	0 (0.0)	0 (0.0)	
IV	5 (31.3)	4 (28.6)	0 (0.0)	
V	10 (62.5)	9 (64.3)	1 (100.0)	
VI	0 (0.0)	1 (7.1)	0 (0.0)	
Grade above V	10 (62.5)	10 (71.4)	1 (100.0)	0.587

*TAWH was graded according to the classification proposed by Dennis et al. [3]: grade III, single abdominal wall muscle disruption; grade IV, complete abdominal wall muscle disruption; grade V, complete abdominal wall muscle disruption with herniation of abdominal contents; and grade VI, open herniation (evisceration)

Abbreviation: LOS, length of stay; ICU, intensive care unit; ISS, Injury Severity Score; TAWH, traumatic abdominal wall hernia

Table 3 shows the characteristics of TAHW repair. Among the 31 patients, 22 (71.0%) underwent operative management, while nine (29.0%) were managed nonoperatively. Among nonoperative repair cases, exploratory laparotomy was performed in four cases for other intra-abdominal indications (12.9%). Of the 22 cases underwent surgical repair for TAWH, 90.9% (*n* = 20) received operative treatment during their initial hospitalization. Among these, 11 patients (55.0%) underwent emergent repair upon admission, while 9 patients (45.0%) underwent delayed or elective repair, either as a planned procedure or during subsequent surgery following an initial non-TAWH operation. The mean time to surgery was 2.3 ± 5.7 days in the emergent repair group and 177.0 ± 107.5 days in the late repair group. All the surgical procedures were performed using an open surgical approach. Primary repair was performed in 20 cases (90.9%), with mesh placement required in only 2 cases (9.1%). Intra-abdominal contamination was present in 8 patients (40.0%) who underwent primary repair, whereas no contamination was present in the mesh placement group. Fisher’s exact test showed no statistically significant difference in intra-abdominal contamination rates between the two groups (*p* = 0.515). Most primary repairs involved muscle and fascial suture repair (*n* = 14, 70.0%) with fixation to the iliac crest (*n* = 5, 25.0%) or rib (*n* = 1, 5.0%) in selected cases. The recurrence rate was 18.2% (*n* = 4), and reoperation performed in 3 cases. Two cases of traumatic inguinal hernia recurred after deep inguinal ring closure in the initial procedure and were later repaired using mesh. One case of recurrent lumbar hernia initially repaired with iliac crest fixation required mesh reinforcement. There was no statistically significant difference among cases with primary repair (*n* = 20); recurrence occurred in 4 cases (20.0%), whereas no recurrence was observed in patients who underwent mesh repair (*n* = 2). No mesh infections were reported, but SSI developed in 4 cases (18.2%). When comparing SSI rates according to intra-abdominal contamination in patients undergoing early surgical repair, SSI occurred in 2 of 12 patients (16.7%) without intra-abdominal contamination and in 2 of 8 cases (25.0%) with intra-abdominal contamination. This difference was not statistically significant (*p* = 1.000).

**Table 3:** Characteristics of patients with hernia repair

Variables	Cases (*n* = 22)
Operative timing, early	20 (90.9)
Emergent repair	11/20 (55.0)
Delayed or elective repair	9/20 (45.0)
Time to repair (days)	8.3 ± 21.6 (1.0)
Early repair	2.3 ± 5.7
Late repair	177.0 ± 107.5
Repair at first laparotomy	18 (81.8)
Approach	
Open	22 (100.0)
Laparoscopic	0 (0.0)
Incision	
Midline	12 (38.7)
Flank	6 (19.4)
Ventral oblique	3 (9.7)
Subcostal	1 (3.2)
Methods	
Primary repair	20 (90.9)
Muscle and fascia suture repair	14 (70.0)
Suture fixation to iliac crest	5 (25.0)
Suture fixation to rib	1 (5.0)
Mesh placement	2 (9.1)
Postoperative complications	8 (36.4)
Recurrence	4 (18.2)
Mesh infection	0 (0.0)

**Fig. 4:**
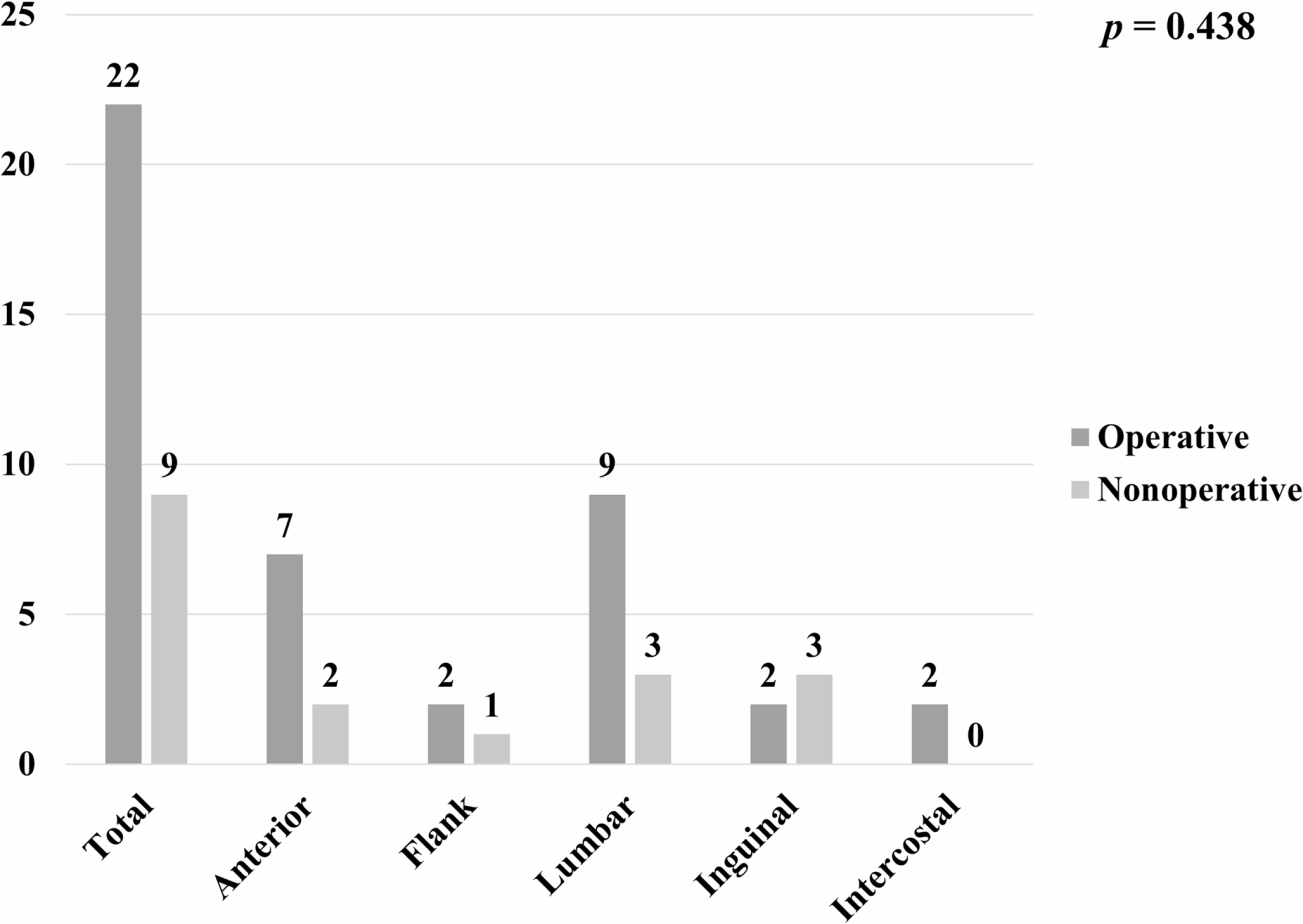
Comparison of operative and nonoperative treatment of TAWH based on hernia location

Figure 4 shows the distribution of hernia locations according to the operative management. No statistically significant difference was observed in the overall distribution between the operative and non-operative groups (*p* = 0.438). Cases in the hernia repair group more frequently had anterior and lumbar hernias than the nonoperative group, without statistical significance (31.8% vs. 22.2%, *p* = 0.689 and 40.9% vs. 33.3%, *p* = 1.000, respectively), while inguinal hernias were more common in the nonoperative group, without statistical significance (9.1% vs. 33.3%, *p* = 0.131).

## Discussion

TAWHs are rare clinical entities, with incidences ranging from 0.17% to 1.5% among blunt trauma admissions [3, 4, 6, 12, 20]. Although a number of single-center reports have described institutional experiences with TAWH, comprehensive analyses and detailed characterization have been relatively limited [3, 4, 7]. In this study, 31 TAWH cases were identified over a 15-year period at a high-volume regional trauma center that was functionally comparable to a Level I trauma facility. The incidence was 0.16%, consistent with prior literature.

Obesity has been proposed as a potential risk factor for TAWH [21, 22], and advanced age or diminished muscle strength are considered predisposing factors for abdominal wall hernias [23–26]. However, TAWHs are reported to occur more frequently in younger males, likely due to increased exposure to high-energy trauma mechanisms, such as MVC [27]. The mean age was 52.2 years, and 64.3% of patients were male, with BMI and SMM within the normal reference range for the Korean population, indicating that constitutional factors, such as sarcopenia or obesity, were not prominent in our sample. These results suggest that the mechanism and energy transfer may play a more decisive role in the development of TAWH than patient-specific factors.

TAWHs are frequently accompanied by concomitant injuries, particularly those involving intra-abdominal structures such as the small bowel and mesentery [11, 28, 29]. Burt et al. [12] reported that 61% of patients with traumatic lumbar hernias sustained intra-abdominal injuries, while Honaker et al. [4] reported that TAWH was accompanied by additional injuries, including abdominal, spinal, or pelvic trauma, in 89.5% of patients. In our study, 89.3% of patients presented with at least one concomitant injury. These included intra-abdominal injuries, such as traumatic hemoperitoneum, bowel injury, solid organ injury, thoracic injuries, orthopedic injuries (including lumbar spine, pelvic, and femur fractures), genitourinary injuries, and vascular injuries. To our knowledge, this is one of the more detailed characterizations of injury patterns associated with TAWH in the relevant literature. These results highlight the importance of thorough evaluation of patients with TAWH, given the substantial overall injury burden.

TAWH is classified into three major types based on the mechanism of injury and size of the abdominal wall defect [30]. Liasis et al. further outlined the historical framework for TAWH classification, incorporating factors such as anatomical location, mechanism of trauma, hernia size, and accident type [13]. However, they did not propose a clinically applicable classification system. In contrast, previous studies have emphasized the importance of biomechanical forces and energy transfer principles in TAWH development, suggesting a more functional classification based on injury dynamics [19]. We applied this biomechanical classification to analyze injury patterns. TAWHs resulting from direct blunt trauma accounted for the majority (53.0%) of cases. High-energy mechanisms causing a sudden rise in intra-abdominal pressure, such as MVCs, were observed in 43.0%. The latter group demonstrated a significantly higher ISS and included one case of evisceration. These findings support the relevance of mechanism-based classifications in predicting injury severity.

A TAWH diagnosis necessitates a careful physical examination and a high index of clinical suspicion. In our study, the most common symptom was localized abdominal pain and tenderness at the hernia site, observed in 64.3% of patients. With increased abdominal CT use in hemodynamically stable patients with blunt trauma, the detection of TAWH has improved, and CT imaging is considered the modality of choice for the diagnosis [3, 7, 27]. Despite this, our findings revealed a notable discrepancy between the imaging evidence and formal radiology reports. Although TAWH were identified on CT in 96.4% of cases, they were explicitly mentioned in the radiology report in only 48.1%, raising concerns about potential under-recognition. These findings underscore the need for trauma surgeons and radiologists to maintain awareness of TAWH, particularly high-energy blunt trauma, despite its rarity.

The optimal timing and technique for TAWH repair remain unclear. Early intervention may facilitate primary fascial closure under reduced tension; however, concerns about contamination often limit the use of synthetic meshes. A recent multicenter study by the Western Trauma Association reported that early repair during index hospitalization was not associated with increased complications or recurrence relative to delayed repair, despite lower mesh use and higher rates of primary closure [31]. Similarly, Liesegang et al. demonstrated that emergency repair yielded favorable early outcomes [32]. In our cohort, early repair was performed in 90.9% of surgically treated patients, with low recurrence and complication rates. Two patients underwent late repair after discharge: one due to late hernia development and the other due to an initially missed diagnosis, despite no concomitant intra-abdominal injury. Both patients underwent mesh repair without subsequent complications. These findings align with growing evidence supporting early repair performed in appropriate clinical settings, particularly in the presence of concurrent intra-abdominal injuries. Nevertheless, late repair may be safely performed in selected patients, particularly when elective mesh use is preferred and urgent surgery is not required.

The TAWH location has been associated with variability in the need for operative intervention. Studies have reported that anterior hernias are more often associated with intra-abdominal injuries requiring surgery, whereas lumbar and flank hernias are frequently managed nonoperatively because of their lower likelihood of concomitant visceral injury [7, 32, 33]. In our cohort, there was no statistically significant difference in the distribution of hernia locations between the operative and nonoperative groups. However, anterior and lumbar hernias were more frequent among surgically treated patients, and inguinal hernias were more common in nonoperative cases, without statistically significant differences, suggesting that while location may influence surgical decision-making, it should not be the sole determinant.

This study had several limitations. The relatively small sample size limits the statistical power of the subgroup analyses. As this was a single-center study, the generalizability may also be restricted. Variability in radiological reporting and clinical documentation could have contributed to the under-recognition of TAWH during the early phases of care. Furthermore, the retrospective nature of the study introduces the potential for diagnostic variability and observer-related bias in CT interpretation despite efforts to minimize such bias through blinded, independent, and consensus-based reviews. Finally, the lack of long-term functional outcomes and a standardized duration of follow-up limits our ability to assess the durability of repair techniques and the clinical impact of recurrence. Multicenter studies with standardized protocols are warranted to validate these findings and guide evidence-based management.

Despite these limitations, this study provides valuable insights into the clinical spectrum, diagnostic challenges, and operative patterns associated with TAWH in an East Asian population with trauma. To our knowledge, this is one of the few single-institution analyses in a non-Western setting that applies standardized grading and classification systems to characterize TAWH with detailed radiologic, clinical, and operative correlations. Our findings reinforce the strong association of TAWH with high-energy mechanisms and concomitant injuries and highlight gaps in radiological reporting. Furthermore, by integrating contemporary CT-based muscle mass analysis, we provided novel data on sarcopenia prevalence and its limited contribution as a risk factor for TAWH. These findings contribute to the growing evidence base and may help refine surgical decision-making in trauma systems.

In this study, TAWH accounted for 0.16% of blunt trauma admissions, and was frequently associated with high-energy mechanisms and intra-abdominal injuries. CT effectively diagnosed TAWH, despite frequent underreporting. Early surgical repair during index hospitalization is feasible and safe, with low recurrence and complication rates. Although only two patients required delayed repair, these cases highlight the potential for late development or missed diagnoses, emphasizing the importance of follow-up. Multicenter studies are warranted to establish standardized diagnostic and management guidelines.

## Supplementary Information

Below is the link to the electronic supplementary material.

Supplementary material 1 (DOCX 172.9 kb)

## Data Availability

The data supporting the findings of this study are available upon request from the corresponding author. The data were not publicly available because of privacy or ethical restrictions.
