# Supplementary material for: Traumatic abdominal wall hernias: A 15-year single-center experience in diagnosis and management
Source: Surg Today. 2025 Nov 19;56(5):753–62. doi: 10.1007/s00595-025-03179-8 (PMC13090226; doi:10.1007/s00595-025-03179-8)
Supplement: Supplementary file 1 — Supplementary material 1 (DOCX 172.9 kb) [file 595_2025_3179_MOESM1_ESM.docx]

**Supplementary Table 1. Clinical characteristics of patients with traumatic abdominal wall hernia**

| **Variables** | **Patients (n = 28)** |
| --- | --- |
| **Abdominal signs on presentation** |  |
| Localized or diffuse abdominal pain | 18 (64.3) |
| Abdominal contusion including seat-belt sign | 8 (28.6) |
| Abdominal wall bulging or swelling | 5 (17.9) |
| Evisceration of abdominal contents | 1 (3.6) |
| None | 5 (17.9) |
| **CT findings** |  |
| Radiological evidence of TAWH | 27 (96.4) |
| TAWH included in radiology report | 13/27 (48.1) |

Abbreviations: CT, computed tomography; TAWH, traumatic abdominal wall hernia.

**Supplementary Table 2. Lesion characteristics of traumatic abdominal wall hernia.**

| **Variables** | **Patients (n = 28)** |
| --- | --- |
| **Laterality** |  |
| Right | 14 (50.0) |
| Left | 10 (35.7) |
| Median | 1 (3.6) |
| Bilateral | 3 (10.7) |
| **Number of locations** |  |
| Single | 25 (89.3) |
| Multiple | 3 (10.7) |
| **Variables** | **Total hernias (n = 31)** |
| **Anatomical locations** |  |
| Anterior | 9 (29.0) |
| Rectus | 4 (12.9) |
| Spigelian | 5 (16.1) |
| Flank | 3 (9.7) |
| Lumbar | 12 (38.7) |
| Inguinal | 5 (16.1) |
| Intercostal | 2 (6.5) |
| **Grade** |  |
| III | 1 (3.2) |
| IV | 9 (29.0) |
| V | 20 (64.5) |
| VI | 1 (3.2) |
| **Herniation contents** | n = 21 |
| Bowel | 15 (48.4) |
| Omentum | 5 (23.8) |
| Mesentery | 1 (4.8) |
| **Defect size (cm), mean ± SD** | 5.3 ± 3.6 |

Abbreviations: SD, standard deviation.


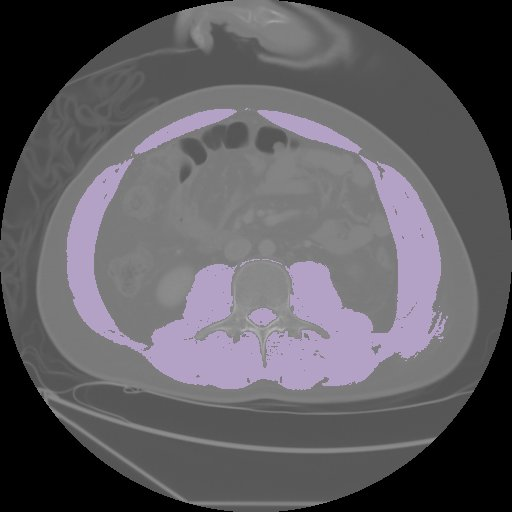


**Supplementary Figure 1.**

Cross-sectional computed tomographic images at the third lumbar vertebral level. Skeletal muscle area was quantified using the threshold values of -29 to 150 Hounsfield units.
